# Neglected Basal Cell Carcinomas in the 21st Century

**DOI:** 10.1155/2011/392151

**Published:** 2010-11-25

**Authors:** Erika Varga, Irma Korom, Zoltán Raskó, Erika Kis, János Varga, Judit Oláh, Lajos Kemény

**Affiliations:** ^1^Department of Dermatology and Allergology, Albert Szent-Györgyi Clinical Center, University of Szeged, Korányi fasor 6, 6720 Szeged, Hungary; ^2^Department of Oral and Maxillofacial Surgery, Albert Szent-Györgyi Clinical Center, University of Szeged, Kálvária sgt. 57, 6725 Szeged, Hungary

## Abstract

Although tumors on the surface of the skin are considered to be easily recognizable, neglected advanced skin neoplasms are encountered even in the 21st century. There can be numerous causes of the delay in the diagnosis: fear of the diagnosis and the treatment, becoming accustomed to a slowly growing tumor, old age, a low social milieu, and an inadequate hygienic culture are among the factors leading some people not to seek medical advice. The treatment of such advanced neoplasms is usually challenging. The therapy of neglected cases demands an individual multidisciplinary approach and teamwork. Basal cell carcinoma (BCC), the most common cutaneous tumor, usually develops in the elderly, grows slowly, and has an extremely low metastatic potential; these factors are suggesting that BCCs might well be the “ideal candidates” for neglected tumors. Five neglected advanced cases of BCC were diagnosed in our dermatological institute between 2000 and 2009. The clinical characteristics and treatment modalities of these neoplasms are discussed, together with the possible causes of the neglect.

## 1. Introduction

Tumors on the surface of the skin are generally visible and considered to be easily recognizable both for health-care professionals and for the patients themselves. However, people with neglected advanced skin neoplasms are still encountered in dermatological practice in the 21st century. There can be numerous causes of the delay in the diagnosis: the person may fear the diagnosis and the treatment or become accustomed to the usually slowly-growing tumor. Old age, a low social milieu, and an inadequate hygienic culture may also be factors explaining why some people are not aware of the significance of a delayed diagnosis. 

Basal cell carcinoma (BCC) is the most common cutaneous tumor and one of the most frequent skin diseases observed by dermatologists [1, 2]. Most of these tumors arise in the head and neck area, particularly in the elderly, and usually grow slowly. The metastatic potential is very low and is mainly detected in association with aggressive or long-standing, large neglected tumors [1–4]. The characteristics of BCCs suggest that they might well be the “ideal candidates” for neglected tumors. During the past 10 years, 5 neglected advanced cases of BCC were diagnosed in our regional center of dermatological oncology.

## 2. Case Reports

The main clinical characteristics of the patients and the treatment applied are listed in Table 1.

**Case 1**

**Figure 1:**
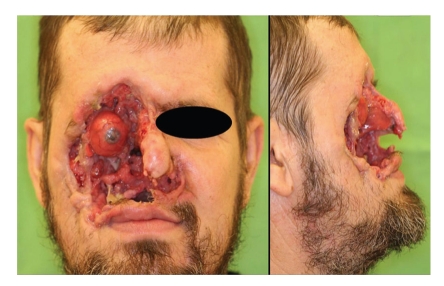
Case 1: A mutilating tumor on the right side of the patient's face.

**Figure 2:**
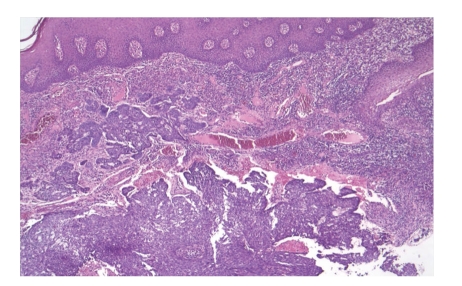
Case 1: The histopathology of the first biopsy specimen confirmed the diagnosis of BCC.

**Figure 3:**
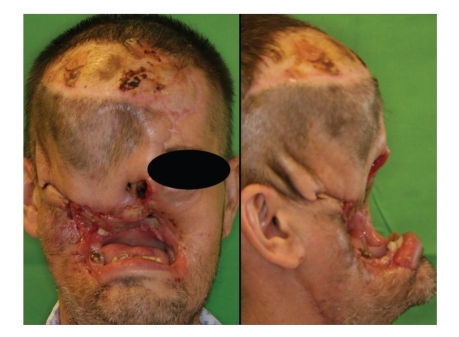
Case 1: The patient is tumor-free, but his face has not been reconstructed functionally or esthetically.

When a 44-year-old male first sought for medical advice in December 2009, he had already had a mutilating, horrifying tumor on the right side of his face for 10 months. He lived alone in a farmhouse in a rural, but quite densely populated area. Two years previously, his own dog had clawed on the right side of the patient's nose, with the injury leaving a scar. Eleven months before his presentation, he had suffered another minor injury in the same region caused by a splinter of wood. A gradually growing, ulcerating lesion had subsequently developed on his face (Figure 1). The clinical diagnosis was BCC, but the possibility of Wegener's granulomatosis also emerged together with a suspicion of leprosy or some other bacterial infection. The CT scan revealed an extensive bone defect: the maxillary sinus had perished, half of the nasal bone and the zygomatic arch were missing, and the external wall of the frontal sinus was involved. As three types of bacteria were identified, intravenous antibiotic treatment was started. Multiple biopsies confirmed the presence of BCC (Figure 2). The tumor was resected at the Oral and Maxillofacial Surgery Department and the defect was covered with a frontotemporal rotation flap and a latissimus dorsi musculocutaneous free flap. In the postoperative period, the patient suffered a right-sided temporal lobe emollition of the brain, resulting in left-sided hemiplegia. The skin layer of the flap necrotized, but the muscular layer remained intact, and healed by secondary epithelialization. After 6 months, recurrences around the mouth necessitated repeated excisions with a forearm fascio-cutaneous free flap reconstruction. This flap necrotized in full thickness and it had to be removed, and the defect was closed in a direct manner. The patient is now tumor-free, but his face has not been reconstructed functionally or esthetically (Figure 3). His nutrition is solved, and physiotherapy has led to a significant improvement in his extremity movements: he can walk alone and use his left hand.

**Case 2**

**Figure 4:**
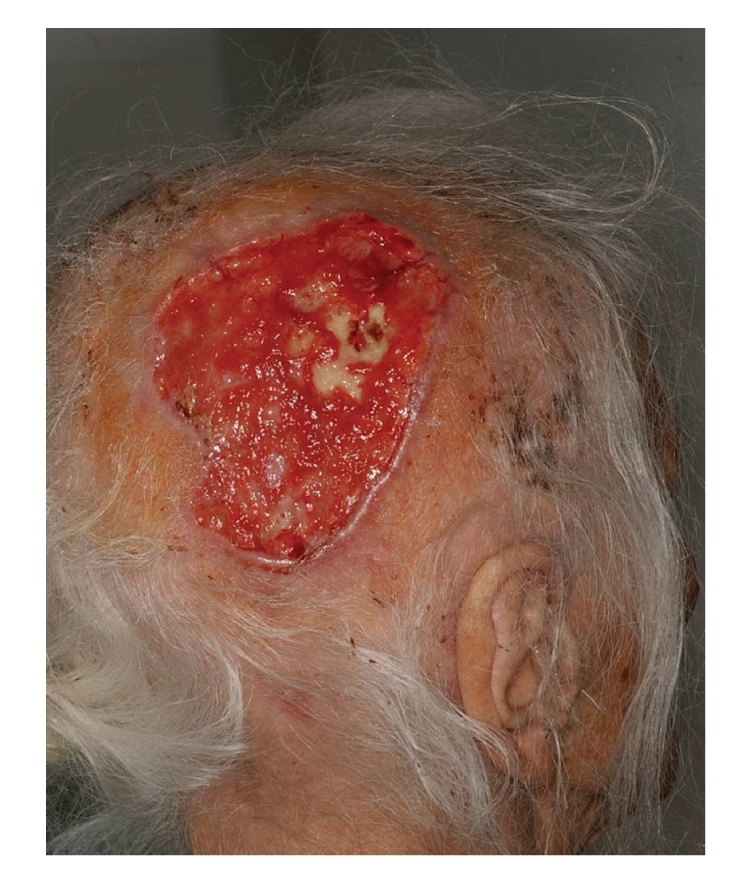
Case 2: An ulcerated tumor in the right parieto-occipital region, with bone destruction.

A 96-year-old female, living in a small town, presented in November 2007 with a 2-year history of a growing tumorous mass in the right inguinal region. The tumor was removed and the histopathology confirmed the presence of squamous cell carcinoma (SCC). At the same time, an ulcerated tumor on the right parieto-occipital region of her head was diagnosed clinically as BCC. Excision or irradiation therapy was planned after the removal of the inguinal tumor, but the patient did not continue the treatment. In 2009, she received neurosurgical treatment and was then referred to dermatology because of her growing cranial tumor (Figure 4). An incisional biopsy from this tumor confirmed the diagnosis of BCC. In view of her age and the size and extent of the tumor, the multidisciplinary oncology team decided on irradiation therapy. This caused the tumor to regress, leaving an ulcer with visible bone at the base. In the meantime, the SCC recurred in the left groin lymph nodes, but no other dissemination was detected and the tumor mass remained stable for several months.

**Case 3**

**Figure 5:**
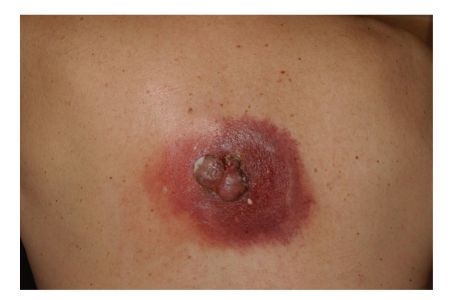
Case 3: An exophytic, ulcerated tumor mass in the middle of the back, with suppurative inflammation and abscess formation.

A 63-year-old man living in an agricultural town had had a small exophytic skin-colored nodule in the middle of his back for many years. This had started to grow rapidly and become ulcerated during the past few months. By his first medical visit in September 2009, suppurative inflammation with abscess formation had developed in the nearby dermis (Figure 5). Consequently, an incisional biopsy and incision of the abscess were initially performed, and systemic antibiotic therapy was administered. The histopathological diagnosis was BCC. Laboratory tests revealed that the patient had previously unrecognized non-insulin-dependent diabetes mellitus. In a second operation, the whole tumor was removed with conservative wound care because of the inflammation. The patient at first participated in regular surgical followup and treatment of the remaining ulcer, but he later failed to appear for the oncology followup.

**Case 4**

**Figure 6:**
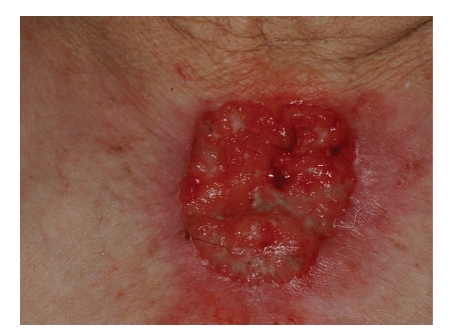
Case 4: An ulcerated tumor on the patient's presternal area.

An 84-year-old male resident in an agricultural village presented in September 2008 with numerous actinic keratoses on his head and neck and with a slowly growing tumorous mass in the presternal area, which he had observed one year previously (Figure 6). The tumor which proved to be a BCC was removed with free surgical margin and the defect was covered by a mesh-graft skin transplant. At the same time, an in situ superficially spreading malignant melanoma in a melanocytic nevus was removed from his back. The patient has currently been under regular followup for 2 years.

**Case 5**

**Figure 7:**
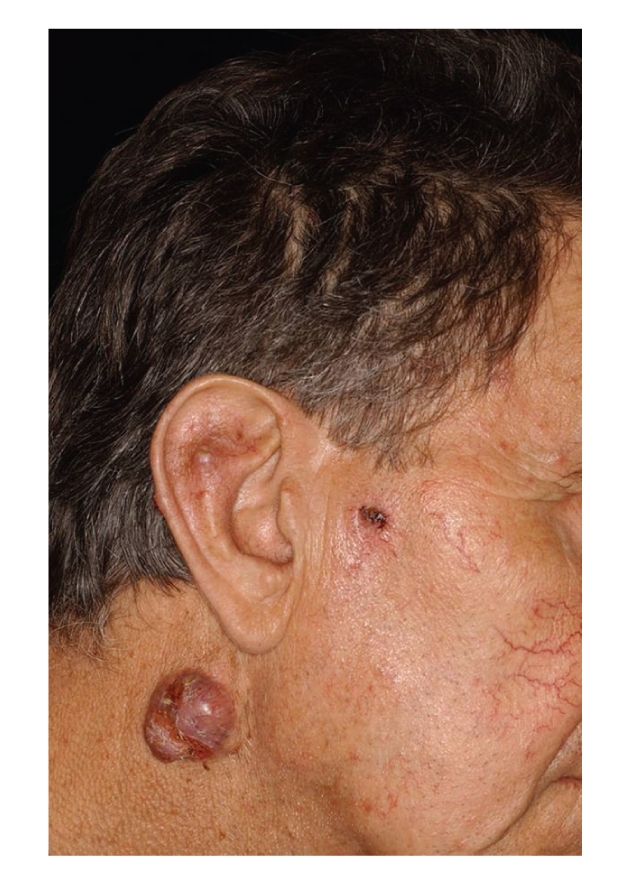
Case 5: A BCC on the right side of the neck and two smaller SCCs in the preauricular region and on the ear.

A 68-year-old male, living in a village, underwent renal transplantation in 1995 and had subsequently received immunosuppressive therapy. A slowly growing tumor developed on the right side of his neck one year prior to his clinical presentation in 2009. Both the clinical and the histopathological diagnosis indicated BCC. Additionally, there were two small SCCs in the preauricular region and on the right ear (Figure 7).

## 3. Discussion

Around one-third of giant BCCs result from patients' neglect [1, 2, 5]. There can be numerous reasons for delay in seeking medical advice. The factors are best documented in malignant melanoma cases [6–8], but nonmelanoma skin cancers and mesenchymal tumors [9–13] are also seen in neglected forms from time to time. A low social milieu, inadequate hygienic culture associated with poverty, and a low level of knowledge about skin tumors may be the explanation in some cases. Patients in these circumstances may not be aware of the possible significance of their growing lesion, though most of our patients live in towns or villages where family members, family doctors or neighbors are easily accessible and media campaigns can reach them. Old age and a slowly growing, not painful neoplasm may also result in a delay in seeking medical advice. The patients may not see properly or not realize the changing and extremely unpleasant clinical picture or they might accept the slowly, but continuously progressing situation. Finally a delay may be caused by an incorrect initial diagnosis, although this occurs mainly with melanocytic tumors [6]. The organ transplanted and/or immunocompromised individuals comprise a special group. In consequence of the immunosuppression, skin tumors are more frequent and more rapidly growing in these individuals. 

It is interesting, that although the patients themselves are more or less aware of the growing tumor, it is usually some external event that finally impels them to seek medical advice, for example, a sudden change in the lesion (bleeding or sudden growth) or encouragement by another person (a family member or friend) [1–3, 6, 7]. A media campaign or a news article may sometimes stimulate a person to visit a physician [6–8]. 

Another challenging question is the treatment of these advanced neoplasms. Numerous possibilities are available for ordinary BCCs: surgical excision, Mohs micrographic surgery, PDT therapy, cryosurgery, immunotherapy, and radiotherapy are the most frequently used techniques [1, 3]. The therapy of neglected cases, however, demands an individual multidisciplinary approach and teamwork. The treatment of choice is often surgery (plastic, craniofacial, or neurosurgery) alone or combined with radiotherapy, with the help of imaging techniques (CT, MRI, and angiography). Reconstruction and a long-term followup are usually needed, with the cooperation of medical experts. Despite the choice of the best possible treatment modalities, a rather unfavorable prognosis and a high recurrence rate are to be anticipated.

## 4. Conclusions

Neglected advanced skin tumors can be encountered even in the 21st century. BCC, the most common cutaneous tumor, usually develops in the elderly, grows slowly, and has an extremely low metastatic potential making it an “ideal candidate” for a neglected tumor. Although there are many possibilities for the treatment of BCCs, the therapy of such neglected cases always demands an individual and multidisciplinary approach and teamwork.

## Figures and Tables

**Table 1:** Data on the neglected BCC patients and their treatment (BCC: basal cell carcinoma, SCC: squamous cell carcinoma).

Case no	Age/sex	Residence	Duration, clinical history	Diagnosis	Treatment
1	44 y male	farm	injury 2 years previously, growing lesion for 10 months	BCC	surgical removal
2	96 y female	town	>2 years	BCC	radiotherapy
3	63 y male	town	several years, rapidly growing for months	BCC with suppurative inflammation	surgical removal
4	84 y male	village	1 year	BCC	surgical removal
5	68 y male	village	1 year, immunosuppressed (renal transplant) patient	neck: BCC face, ear: SCC	surgical removal
